# Intra-Articular Steroid Injection for Patients with Hip Osteoarthritis: A Systematic Review and Meta-Analysis

**DOI:** 10.1155/2020/6320154

**Published:** 2020-02-24

**Authors:** Hui-Ming Zhong, Guang-Feng Zhao, Tiao Lin, Xin-Xin Zhang, Xin-Yu Li, Jun-Fan Lin, Si-Qi Zhao, Zhi-Jun Pan

**Affiliations:** ^1^Department of Emergency, Second Affiliated Hospital, Zhejiang University School of Medicine, Zhejiang University, Hangzhou 310009, China; ^2^Department of Musculoskeletal Oncology, First Affiliated Hospital of Sun Yat-sen University, 58#, Zhongshan 2 Road, Guangzhou 510080, China; ^3^Zhongshan School of Medicine, Sun Yat-sen University, 76#, Zhongshan 2 Road, Guangzhou 510080, China

## Abstract

**Purpose:**

The aim of this current review was to confirm the efficacy of intra-articular steroid therapy (IAST) for patients with hip osteoarthritis (OA) and discuss the duration and influential factors of IAST.

**Methods:**

Online databases (Medline, EMBASE, and Web of Science) were searched from inception to May 2019. Both randomized controlled trials (RCTs) and noncontrolled trials assessing the efficacy of hip IAST on pain were included. Common demographics data were extracted using a standardized form. Quality was assessed on the basis of Oxford Centre for Evidence-Based Medicine 2011 Levels of Evidence.

**Results:**

12 trials met the inclusion criteria. According to data from individual trials, IAST had significant efficacy on hip OA in both immediate and delay pain reduction, which persisted up to 12 weeks after IAST. The influences of the baseline severity of hip OA or synovitis and injection dose or volume on the clinical outcome of IAST were still controversial. The IAST appeared to be well tolerant by most of the participants.

**Conclusion:**

IAST was proved to be an efficacious therapy in both immediate and delay pain reduction for hip OA patients within 12 weeks. The longer follow-up data of efficacy and safety and potentially influential factors are still unclear and needed further confirmation.

## 1. Introduction

Hip osteoarthritis (OA), involving major structural changes of the joint, is one of the most common articular diseases, and the prevalence increases with age [1]. The hip pain and functional disorders in this population result in great troubles in their daily activities and life quality [2].

To reduce pain and restore function, the main therapy methods for hip OA comprise surgical interventions such as total hip replacement (THR) operation and nonsurgical interventions such as exercise therapy [3] and medication therapy [4]. Patients with THR might need a revision of their hip replacement in the future, while nonsurgical interventions before surgery with pain reduction are supposed to delay primary replacement or avoid revision surgery [5].

Intra-articular steroid therapy (IAST) is likely to be an available candidate to supply a local analgesic effect for OA patients. However, published data related to IAST on hip OA patients were varied and the efficacy of pain reduction in the long term is rarely evidenced. The rationale of applying IAST is mainly based on the evidence from knee OA patients. A previous systematic review [6] reported an efficacious pain reduction with IAST in hip OA patients, on which the treatment came into effect one week after injection, and the beneficial efficacy on pain relief decreased thereafter. But this review only summarized five studies with 346 participants during a short-term follow-up, rendering that it was still unclear how long the efficacy would persist and what are the impacts influential factors, such as the injection dose, the severity of OA, or other predictors, would have on the clinical outcomes.

The objectives of this comprehensive review were to confirm the efficacy of IAST for patients with hip OA and discuss the duration and influential factors of the IAST. The safety profiles were summarized as well.

## 2. Methods

### 2.1. Literature Search

Online databases (Medline, EMBASE, and Web of Science) were searched from inception to May 2019 with the following terms in Boolean logic: “hip osteoarthritis AND intra-articular injection AND (steroids OR methylprednisolone OR triamcinolone OR betamethasone).” There were no restrictions on language, age, or publication dates. Randomized controlled trials (RCTs), noncontrolled clinic trials, and cohort and case-control studies were all applied. We also searched references of review articles for access to other available studies and reduce possible omission.

### 2.2. Study Selection and Quality Assessment

This selection included studies that (a) reported on patients suffering pain caused by hip OA, which was diagnosed based on ACR criteria and/or radiographic evidence, (b) contained intervention group(s) in which patients received IAST, (c) consisted of IAST and placebo groups in RCTs or IAST groups with different regimes in noncontrolled trials, and (d) reported usable pain reduction outcomes. The exclusion criteria were (a) protocols or reviews, (b) animal studies, and (c) those without usable data. After duplication was checked and excluded, 2 reviewers (LXY and LJF) ruled out those ineligible articles by title and abstract screening. Then, 2 reviewers (ZSQ and LXY) evaluated the qualification of the trials for inclusion by reading the full articles, respectively, and the disagreements were resolved by discussion. ZSQ and LJF also rated the quality of included studies according to Oxford Centre for Evidence-Based Medicine 2011 Levels of Evidence [7]. We also used the criteria of the Grading of Recommendations Assessment, Development and Evaluation (GRADE) to evaluate the quality of evidence [8]. When disagreements occur, we would discuss to seek resolutions [9].

### 2.3. Extraction and Analysis of Data

In each included trial, its study design, sample sizes of steroid and control groups, patients' age and gender, OA definition, patient inclusion criteria, medication, and guidance of injection were extracted under standardized forms by ZHM and ZXX.

The pain outcome measurements of each included trial in baseline and different intervals after injection, duration of follow-up, numbers of losses to follow-up, and reported side effects were also extracted and summarized.

The pain scores of patients after treatments are our primary outcome. We firstly summarized the reported endpoint data of these trials, and if the primary data were unavailable, we measured them from the figures to maximize data availability. When an article reported pain data on more than one pain score, we were likely to extract the pain score which was highest on the list of the suggested hierarchy of continuous pain-related outcomes used in the meta-analysis [10]. Besides, considering the same patient cohort was measured in baseline and different time points in one trial, when the standard deviation (SD) of some follow-up time points was available, the SD of the baseline data was used for the time-point SD [11].

Considering the varied units of the pain score applied in each RCT, the pain scores were converted to standardized mean differences (SMDs) with the help of the software program Review Manager (RevMan 5.3, Nordic Cochrane Centre, Copenhagen, Denmark, provided by the Cochrane Collaboration), which was also used to generate forest plot graphs of meta-analysis.

For the follow-up duration that varied among the included RCTs, we divided the outcomes into 3 time intervals: 1-2 weeks, 3–4 weeks, and 8–12 weeks. In this case, we can make maximum use of all reported data to show the time-fashion efficacy of IAST for hip OA patients. Long-term results were defined by the reported pain score ≥8 weeks after IAST.

We evaluated the heterogeneity of included RCTs statistically by the *χ*^2^ test on *N* − 1 degrees of freedom (*p* < 0.1 indicated significance) and calculated the ratio of the consistency among those included studies by calculating the inconsistency *I*^2^ using the formula ((*Q* − df)/*Q*) × 100% (*Q*: *χ*^2^ statistic and df: degrees of freedom). We chose the random-effects model to combine different studies' outcomes when the *I*^2^ value was over 50%, which represented a significant inconsistency; otherwise, the fixed-effects model was chosen.

As only 5 RCTs were included, we could not perform sensitivity analysis by funnel plot analysis and meta-regression analysis, and we omitted each individual trial to test the stability of the main outcomes.

Outcomes from the remaining studies which were unable to be pooled by meta-analysis were also summarized, reflecting the impacts contributed by factors such as the injection dose and the severity of OA.

## 3. Results

### 3.1. Selection of Studies

The literature search yielded 301 records potentially relevant to the study question, of which 240 publications were excluded by filtering through clinical trials and duplication checking and 38 were excluded based on the titles and abstracts. For the remaining 23 records, full-text articles were read and 12 records were excluded for not containing useful information. From the references of the included study [12], we found one trial [13] that met the inclusion criteria. Overall, there were 12 trials with 1504 patients included.

### 3.2. Information on Included Trials

The main information on 12 included studies is included in Table 1. The data from 5 studies [11, 12, 14–16] with available control groups were combined in a meta-analysis manner. 4 are randomized control trials (RCTs) containing placebo (local anaesthetic or normal saline or sham injection) groups [11, 14–16], of which two trials just performed head-to-head comparisons between IAST and placebo groups [15, 16], while the other two were multiple-comparison studies comprising steroid, hyaluronic acid, and placebo groups [11, 14]. And another one was the case-control trial [12], which also contained steroid and control groups and was included in the meta-analysis.

Excepting these 4 placebo-control RCTs, another RCT [17] was not able to be synthesized into meta-analysis but the results were described and summarized, instead. All injections were performed under X-ray, ultrasound, or fluoroscopic guidance. And the follow-up period of the included studies for meta-analysis [11, 12, 14–16] was all within 3 months (12 weeks).

All the other studies performed IAST in symptomatic hip OA patients. And they reported either short-term (within 8 weeks after injection) or long-term (≥8 weeks after injection) follow-up.

### 3.3. Methodological Quality

As presented in Table 1, the quality of included studies was defined as different levels of evidence based on Oxford Centre for Evidence-Based Medicine 2011 Levels of Evidence [7].

The evidence quality of the 5 included RCTs [11, 14–17] was considered level 2. Two trials' randomization using computer-generated numbers was considered to be adequate [14, 16], and three studies' allocation concealment was stated [14–16]. Description of withdrawals and dropouts [11, 14, 16, 17] was reported in four articles, and ITT analysis [11, 14, 15] was reported in three articles, respectively. All included RCTs were double-blinded, and selective reporting was not found in any of the trials.

Within the other 7 studies, most of them had noncontrolled design. Nevertheless, there were two controlled cohort trials [18, 19], comparing different doses or volumes of IAST, which achieved level “3” evidence. And another trial [12] was a case-control trial which was also rated as level “3” evidence. The other three trials which are case-series trials [13, 20, 21] were rated as level “4” evidence, in which two trials were retrospective [20, 21]. The last trial [22] was an observational study which achieved level “2” evidence.

### 3.4. Efficacious Pain Reduction within 12 Weeks and Unclear Efficacy in Long-Term Outcomes

The most immediate effect of pain alleviation was reported by Deshmukh [20], which studied 217 patients with fluoroscopic-guided IAST of 5 ml of 0.5% bupivacaine and 1 ml (80 mg) methylprednisolone (MP). Just 15–20 minutes following the procedure, the authors found 68.2% of patients (148/217) got an immediate positive response (patients with more than 50% reduction outcome on VAS scores were defined as positive responders). And their two-week results still presented 71.4% (155/217) positive responders.

Another study containing 40 patients by Micu et al. [12] adopted IAST of 8 mg betamethasone and 2 ml lidocaine 1% plus 0.5 ml air with ultrasound guidance. Four weeks after treatment, this study showed not only a dramatic pain reduction compared to baseline (−66.1%, *p* < 0.001) but also the pain alleviation that persisted at 12 weeks after injection (−55.2%, *p* < 0.001).

Another trial by Margules et al. [13], adopting fluoroscopy-guided injection of 40 mg/mL of triamcinolone acetonide (TAC), suggested 198 of 510 (38.8%) patients still responded positively 8 weeks after the injection.

Similarly, Walter et al. [21] studied IAST with injection of 80/40 mg of TAC (40 mg/ml) and 3/4 ml of 0.5% ropivacaine in 113 patients. In this study, however, the pain score measured by the patient-reported EuroQol 5-domain visual analog scale (EQ5D-VAS) showed insignificant improvement (short term (<8 weeks): EQ5D-VAS = 1 ± 18.32, *p*=0.915; long term (>8 weeks): EQ5D-VAS = 0.25 ± 20.58, *p*=0.455).

The 5 included trials for meta-analysis further confirmed the short-term efficacy of IAST on pain relief of hip OA patients.

**Figure 1:**
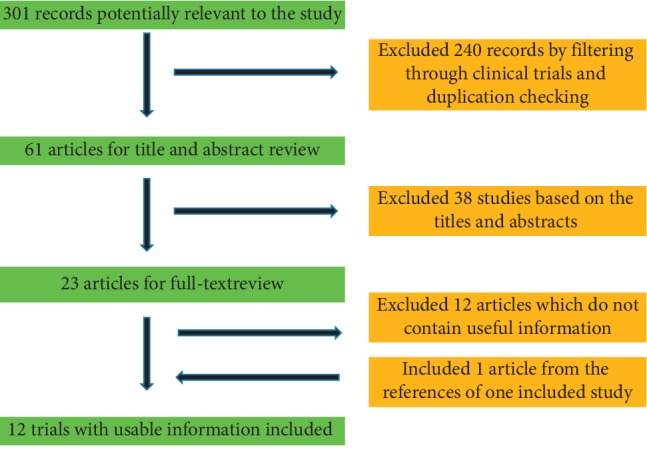
A flow diagram demonstrating the method of article selection for clinical study inclusion.

The pooled analysis of 5 studies [11, 12, 14–16] demonstrated that the steroids were more efficacious than placebo control in pain relief at all three different time intervals. The pain scores in different groups were converted to standard mean differences (SMDs), and the combined results are shown in Figure 1 (1-2 weeks: 2 studies, SMD (95% CI): −1.58 [−3.42, 0.26], *p*=0.09, *I*^2^ = 93%; 3–4 weeks: 4 studies, SMD (95% CI): −1.93 [−3.34, −0.52], *p*=0.007, *I*^2^ = 95%; 8–12 weeks: 5 studies, SMD (95% CI): −1.77 [−2.94, −0.61], *p*=0.003, *I*^2^ = 94%).

**Figure 2:**
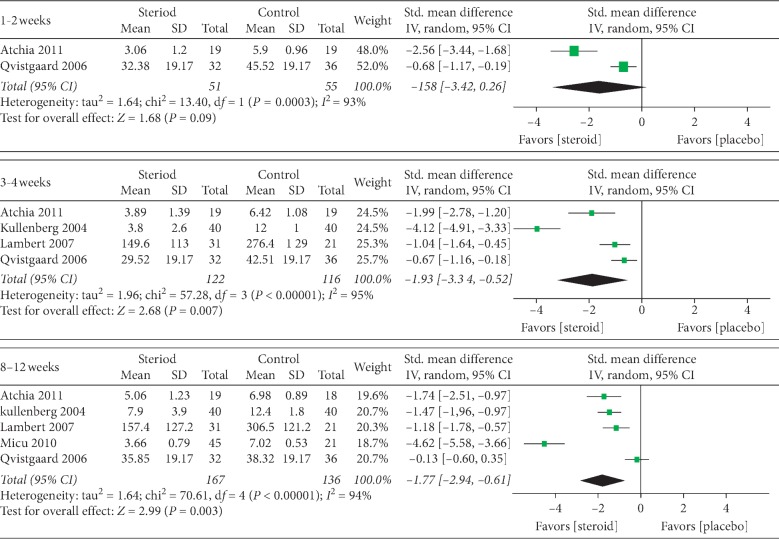
Forest plots of the pain score at different time intervals.

Among these 5 studies, the 12 weeks' data from Micu's study [12] turned out to be an outlier in the forest plot (Figure 2). Nevertheless, the sensitivity analysis suggested that the overall effect size was not substantially affected when the very single study was deleted (before deleting: *Z* = 2.99, *p* = 0.003; after deleting: *Z* = 2.92, *p* = 0.003). This trial which presented as an outlier might be explained by its nonrandomized study design.

Furthermore, the four RCTs also showed a relief of pain in steroid groups at different time intervals compared to the baseline. The effect on pain reduction appeared to be initially large but decreased over time (Table 2).

Another RCT by Flanagan et al. [17] also reported significant pain decrements at four weeks and 12 weeks after IAST of 10 ml of bupivacaine plus 20 mg of TAC. Specifically, they totally reported 9 positive responders in week 4 and 4 positive responders in week 8 after injection. Also, Flanagan's report extended the follow-up time till 48 weeks after treatment, which showed only 3 positive responders in week 24, 2 in week 36, and 1 in week 48.

These results indicated that IAST was an efficacious treatment for reducing hip pain within 12 weeks, although the effect decreased over time. And there is still rare evidence on its efficacy in a longer follow-up.

#### 3.4.1. Controversial Impacts of Radiographic Severity of OA were Found on IAST Efficacy

Deshmukh [17] reported that the advanced hip OA patients were with a greater tendency to exhibit immediate and delayed pain alleviation compared to mild ones. In contrast to this, 8 weeks after IAST with 40 mg/mL of TAC, patients in the group with less severity of OA had more positive response on pain relief (positive responders: severe group: 21/234, 9%; moderate group: 131/226, 58%; mild group: 46/51, 90%) in Margules's study [13]. Nevertheless, Subedi [22] did not present positive relation of OA severity with the pain reduction after IAST. The authors divided 100 patients into four groups based on the hip OA severity and then subjected all the groups to IAST with 80 mg MP and 10 mL bupivacaine. 6–8 weeks after injection, 82% of patients (82/100) reported obvious pain improvement compared to baseline (*p* < 0.01) but nothing related to the categories in OA severity (*p*=0.51).

Furthermore, Lambert et al. [16], Qvistgaard et al. [11], and Robinson et al. [18] also mentioned that pain reduction after IAST was independent of the severity of hip OA at the baseline, although the data were not shown in their results.

The varied results of the included studies might require further study to confirm the influence of the severity of OA on IAST pain reduction.

#### 3.4.2. No Evidence Indicated Severity of Synovitis Has an Impact on Pain Reduction of IAST

Atchia et al. [14] reported the responders' number in the IAST group at 1, 4, and 8 weeks after injection with or without the presence of synovitis, and the results showed that synovitis was proved to be the single predictor of IAST response in weeks 4 and 8 (Fisher's exact test between numbers of responders of synovitis and no-synovitis groups, weeks 4 and 8: *p*=0.04).

However, another trial by Micu et al. [12] divided all 40 patients (with 45 hips) into two groups based on the severity of synovitis, which were called subgroup 75% and subgroup 25% (regression of capsule distention was evident in 34 hips, which was defined as subgroup 75%. And the remaining 11 hips, whose distention of the capsule persisted >8 mm and >2 mm side difference, were defined as subgroup 25%). After receiving the same IAST, the outcomes of VAS scores indicated that both groups had a significant effect on pain reduction within three months (subgroup 75%: 1 and 3 months: *p* < 0.001; subgroup 25%: 1 month: *p* < 0.001, 3 months: *p*=0.003). However, the pain relief was not associated with the severity of synovitis.

The varied results above indicated that the impact the severity of synovitis had on the IAST pain reduction was still unclear.

#### 3.4.3. Dose of Steroids Had Insignificant Effects on the Pain Reduction Outcomes

Robinson et al. [18] studied 120 patients which received ultrasound-guided injection of either 40 mg MP or 80 mg MP and both with 3-4 ml 0.5% bupivacaine. The two groups turned out to both have beneficial effects on pain reduction in week 6 (40 mg: pain score reduction from 12 (baseline) to 10, *p* < 0.001; 80 mg: pain score from 12 (baseline) to 8, *p* < 0.001), while only the effect of the 80 mg group lasted in week 12 (pain score reduction from 12 (baseline) to 10, *p* = 0.002).

In accordance with the above study, in Walter's study [21], all 113 patients received an injection containing either 40 mg or 80 mg of TAC, and the Fisher test showed no significant association among patients with different triamcinolone dose groups (*p*=0.818).

#### 3.4.4. Injection Volume Had No Influence on the Pain Reduction of IAST

Young et al. [19] studied 118 patients which received fluoroscopy-guided injection of either 40 mg TAC and 2 ml bupivacaine or a larger total volume by adding 6 ml of sterile water; after 3 months, treatment patients were evaluated by WOMAC scores. And the outcome indicated that patients of the two groups achieved 28% pain reduction compared to baseline (*p* < 0.001), but there are no differences between the two groups (*p*=0.95).

#### 3.4.5. IAST Is Safe and Well Tolerant by Most Hip OA Patients

The patients' number of losses to follow-up, possible adverse events, and side effects reported by included trials are included in Table 2.

Generally, the IAST was well tolerant by most of the participants, and no serious side effects were reported in all included studies. Specifically, Lambert et al. [16] reported that deep vein thrombosis occurred in one patient of the IAST group 3 months after injection; one patient in the placebo group and 3 patients in the IAST group experienced worse pain after the efficacy of local anaesthetic.

In the trial by Young et al. [19], five slight side effects were reported during their follow-up, where two patients experienced a transient increase in pain, one had soft-tissue swelling, one had facial flush, and one had transient hyperglycaemia in a type I diabetic.

## 4. Discussion

The recommendation of IAST for OA in the current practice is still controversial due to the methodological limitations in the currently available literature [23]. The previous systematic review concluded that hip IAST may provide clinically significant but short-term persisted pain reduction in patients with hip OA. However, this review only contained 5 trials with 345 patients, which included only a small sample size to obtain comprehensive results. Moreover, there was only one time point (8 weeks) discussed in this meta-analysis. Furthermore, this previous review showed also lack of extensive discussion on the influential factors of IAST efficacy.

In comparison with this review, our current review utilized a systematic and comprehensive search strategy to combine the maximum available data, which contained 12 relevant studies involving 1504 participants in total. Moreover, we converted the varied outcome measures into standardized mean differences and pooled the data for three time intervals (1-2 weeks, 3–4 weeks, and 8–12 weeks), respectively, to analyze the therapeutic results, which were quantitative and highly informative in assessing the magnitude of IAST efficacy and provided a clearer therapeutic response mode of the IAST.

Thus, the evidence from this current review indicated that IAST on hip OA patients had significant efficacy on both immediate and delayed pain relief until 12 weeks after injection, though the efficacy was decreased over time.

In addition, based on the current evidence, the longer follow-up data of efficacy are still rarely reported. Walter et al. [21] failed to demonstrate significant outcomes on pain reduction at both immediate and delayed intervals up to 6 months after injection. The unexpected results reported by the study might be explained by several reasons, including limited patient recruitment, and various injection techniques. Its unique outcome measurement might be partly the reason, which was more general for measurement of quality of life, while other studies mostly utilized the outcome instrument specifically designed to evaluate the therapeutic effect of osteoarthritis.

Also, we summarized the potentially influential factors on the efficacy occurrence and persistence of IAST in the included studies, which are rarely summarized in the previous review [6].

Some last updated reports proved that patients with severe knee or hip pain at baseline derived more benefit from IAST at short-term follow-up than those with less severe pain at baseline [24]. In Hirsch's review [25], no significant differences among groups with different severity of synovitis were reported with respect to the primary outcome measure of a numerical rating scale for worst pain, and neither steroid doses (40–80 mg MP) nor the radiographic grade of OA detected any association between IAST and outcomes, which was consistent with this current review. The uncertainties of predictors might be resulted from the great heterogeneity in patients' age, gender, the grade of ACR criteria, choice of the pain outcome extracted, and injection guidance among the available studies and required further clarification by future high-quality large-scale RCTs.

For now, the current guidelines provided by Osteoarthritis Research Society International (OARSI) [26] suggested that IAST should be mostly indicated for patients who suffer moderate to severe pain and do not respond satisfactorily to oral analgesic/anti-inflammatory agents or patients who present with local inflammation. Thus, the severity of OA and response to oral medication should be still under consideration before IAST for most patients.

In the current review, the IAST is also proved to be a safe procedure. Of all 1504 patients, only a few participants were reported to withdraw for side effects, and most fluoroscopic- or ultrasound-guided hip IAST procedures were very well tolerated by participants. However, whether the injection would influence the infection prevalence after the future THR is still under caution [27, 28] and needs to be further studied.

In the GRADE system of rating quality of evidence [8], the evidence quality of RCTs was high at the beginning but was downgraded by four categories of limitations (Table 3). The 95% confidence interval of the SMD in 1- to 2-week outcomes containing the zero line might be considered imprecision and accounted for the downgraded quality of evidence; furthermore, one case-control trial was included into the pooled analysis of 8- to 12-week results which led to a serious limitation. Nevertheless, even substantial heterogeneities can be found across the included trials, which consistently favor steroid over control, we did not downgrade the evidence by inconsistency. The overall strength of references is therefore moderate in intervals of 1-2 weeks and 8–12 weeks and high in the interval of 3-4 weeks (Table 3).

Potential limitations of our review included the large portion of studies with poor quality, which were commonly rated as only level “4” evidence based on the Oxford Centre for Evidence-Based Medicine 2011 Levels of Evidence [7]. Moreover, the lack of patients from primary care units of included studies potentially resulted in selection bias because patients recruited from secondary care units usually had a greater portion of severe hip OA.

In conclusion, this current review provides evidence of applying IAST for hip OA patients to efficaciously improve hip pain up to 12 weeks. The longer follow-up data of efficacy and safety and potentially influential factors are still unclear and need further confirmation.

Since the pooled data did not address the long-term benefits and risks, it is imperative to perform a sufficiently sized and methodologically sound RCT for establishing the definite role of IAST in hip OA. Until such a trial is performed, the current evidence regarding the use of IAST for hip OA is moderate.

## Figures and Tables

**Table 1:** Characteristics of included trials.

Author/year	Study design (level of evidence^*∗*^)	Sample size (steroid/control)	Age (mean ± SD, years) (no. of males/females)	Follow-up, weeks	Loss to follow-up (steroid/control)	Inclusion criteria	Injection	Guidance
Atchia/2011	RCT Steroid/HA/PLB (2)	38 (19/19)	PLB: 70 ± 10 (7/12) Steroid: 67 ± 7 (11/8)	1, 4, 8	Before week 8: 0/0 Week 8: 0/1	(1) Aged over 50 years (2) Primary unilateral hip OA (3) Pain duration was more than a month or listed for elective THR	Single injection: PLB: 3 ml NS; steroid: 3 ml/120 mg Depo-Medrone	Ultrasound

Kullenberg/2004	RCT Steroid/LA (2)	80 (40/40)	Local analgesic: 72.7 ± 6.4 Steroid: 67.3 ± 7.7	3, 12	Before week 12: NA Week 12: 40/40	(1) Pain duration of more than 4 weeks and pain of VAS score > 3 (2) Ahlbäck grade was 2 or worse and joint space narrowing with cartilage destruction of 50% or worse	Single injection: steroid: 80 mg TAC; local analgesic: 1% mepivacaine	Fluoroscopy

Lambert/2007	RCT Steroid/placebo (2)	52 (31/21)	PLB: 56.9 ± 11(11/10) Steroid: 65.6 ± 11(10/21)	4, 8, 12	Week 8: 0/2 Week 12: 11/15 Week 24: 15/18	(1) Aged above 40 years (2) Hip OA as defined by the ACR criteria and radiographic changes (3) Symptoms lasted for at least 6 months (4) Resistance to conventional therapy	Single injection: PLB: 2 ml NS + 10 mg bupivacaine; steroid: 40 mg THA + 10 mg bupivacaine	Fluoroscopy

Qvistgaard/2006	RCT Steroid/HA/placebo (2)	68 (32/36)	PLB: 64 ± 11(14/22) Steroid: 69 ± 9(9/23)	2, 4, 12	Week 4: 2/1 Week 12: 6/3	(1) Aged above 18 years (2) Hip OA as defined by the ACR criteria and radiographic changes (3) Stable medication for at least 3 weeks before inclusion	Three injections with 14-day intervals: PLB: 2 ml NS; steroid: 1 ml/40 mg Depo-Medrone, followed by two sham injections	Ultrasound

Micu/2010	Case-control trial (3)	61 (40/21) 40 patients with 45 hips	62.78 ± 8.16	12	NA	(1) Hip OA with synovitis as defined by the ACR criteria (2) Refractory to conventional therapy for 2 months	8 mg of betamethasone, 2 ml of lidocaine 1%, and 0.5 ml of air	Ultrasound

Flanagan/1988	RCT Steroid/LA/NS (2)	35 (12/23)	Age range: 46–79 (7/28)	4, 8, 24, 36, 48	Week 4: 0/0 Week 8: 0/1 Week 24: 9/11 Week 36: 10/19 Week 48: 11/21	Patients awaiting THR for osteoarthritis	Single injection: LA: 10 ml bupivacaine 0.5%; steroid: 10 ml of bupivacaine plus 20 mg of triamcinolone; NS: 10 ml of normal saline	X-ray

Margules/2001	Case-series trial (4)	510	NA	NA	NA	(1) Patients who did not experience improvement with analgesics or physical therapy (2) Patients who could not tolerate surgery	40 mg/cm^3^ of TAC	Fluoroscopy

Deshmukh/2011	Retrospective case-series trial (4)	217	NA	2	NA	Patients with a clinical diagnosis of hip OA (Kellgren–Lawrence scale)	5 ml of 0.5% bupivacaine and 1 ml (80 mg) MP	Fluoroscopy

Robinson/2007	Nonrandomized controlled cohort trial (3)	40 mg: 75	40 mg: 63.28 (15/60)	12	NA	Patients with a clinical diagnosis of hip OA (Kellgren–Lawrence scale) and symptoms with duration greater than 4 months	40 mg or 80 mg MP and 3–4 ml 0.5% bupivacaine	Fluoroscopy
		80 mg: 45	80 mg: 65.64 (15/30)					

Subedi/2015	Observational study (2)	100	58 (36/64)	6–8	NA	NA	80 mg MP (Depo-Medrone) and 10 ml bupivacaine	Fluoroscopy

Walter/2019	Retrospective case-series trial (4)	113	59 ± 13.7 (36/77)	24	13	Patients with radiographic hip osteoarthritis	80 mg (or 40 mg) of triamcinolone (40 mg/ml) and 3 ml (or 4 ml) of 0.5% ropivacaine	Ultrasound or fluoroscopy

Young/2012	Controlled cohort trial (3)	Low volume: 55 High volume: 55	Low volume: 62 (27/32) High volume: 68 (19/40)	12	Low volume: NA High volume: 6	NA	Low volume: 40 mg triamcinolone and 2 ml bupivacaine High volume: additional 6 ml of sterile water	Fluoroscopy

SD: standard deviation; RCT: randomized controlled trial; HA: hyaluronic acid; PLB: placebo; OA: osteoarthritis; NS: normal saline; VAS: visual analog scale; TAC: triamcinolone acetonide; ACR: American College of Rheumatology; THA: triamcinolone hexacetonide; NA: not available; THR: total hip replacement; LA: local analgesia; MP: methylprednisolone. ^*∗*^The level of evidence of each study was rated on the basis of Oxford Centre for Evidence-Based Medicine 2011 Levels of Evidence.

**Table 2:** Results of the included trials.

Author/year	Pain outcome extracted	Hip pain			Side effects	Potential predictors	Conclusion
		Baseline	Short term <8 weeks	Long term ≥8 weeks			
Atchia/2011	NRS: worst pain^#^	Placebo: 6.55 ± 0.68 Steroid: 5.99 ± 0.62	1 week: placebo: 5.9 ± 0.96; steroid: 3.06 ± 1.2 (*p* < 0.001) 4 weeks: placebo: 6.42 ± 1.08; steroid: 3.89 ± 1.39 (*p*=0.01)	8 weeks: placebo: 6.98 ± 0.89; steroid: 5.06 ± 1.23 (*p*=0.06)	NA	(1) Synovitis (2) Changes in medications	(1) IAST is highly efficacious in pain relief (2) Synovitis is the single predictor in weeks 4 and 8 (3) No differences between NSAIDs and paracetamol or paracetamol/weak opioid groups during the trial

Kullenberg/2004	Total VAS	Placebo: NA Steroid: 12.2 ± 2.2	3 weeks: placebo: 12 ± 1; steroid: 3.8 ± 2.6 (*p* < 0.001)	12 weeks: placebo: 12.4 ± 1.8; steroid: 7.9 ± 3.9 (*p* < 0.01)	NA	NA	IAST might improve pain and range of motion

Lambert/2007	WOMAC pain scores	Placebo: 314.3 ± 76.2 Steroid: 310.1 ± 54.6	1 month: placebo: 276.4 ± 129; steroid: 149.6 ± 113 (*p*=0.0005)	2 months: placebo: 306.5 ± 121.2; steroid: 157.4 ± 127.2 (*p* < 0.0001)	One deep vein thrombosis at 3 months in the steroid group; one patient in the placebo group and 3 patients in the steroid group reported rebound pain	(1) Age (2) Severity of hip OA	(1) IAST is an effective treatment in pain relief (2) The efficacy of IAST remained significant with age as a covariate (3) No association between the severity of OA and the responder status

Qvistgaard/2006	VAS (walking pain)^#^	Placebo: 42.4 ± 19.17 Steroid: 44.0 ± 19.17	2 weeks: placebo: 45.52 ± 19.17; steroid: 32.38 ± 19.17 (*p*=0.006) 4 weeks: placebo: 42.51 ± 19.17; steroid: 29.52 ± 19.17 (*p*=0.006)	12 weeks: placebo: 38.32 ± 19.17; steroid: 35.85 ± 19.17 (*p*=0.58)	NA	(1) Degree of OA (2) Effusion in the joint (3) Age	(1) IAST provided significant improvement in pain within 3 mo (2) Not related to the severity of OA, effusion in the joint, and age

Micu/2010	VAS (walking pain)	Control: 8.66 ± 0.79 Steroid: 8.17 ± 0.86	1 month: control: NA; steroid: 2.77 ± 0.79 (*p* < 0.001 vs. baseline)	3 months: control: 7.02 ± 0.53; steroid: 3.66 ± 0.79 (*p* < 0.001 vs. baseline)	Transient facial rash was present in 16 patients during the first 24–48 h after injection	Severity of synovitis	(1) IAST is an efficacious and safe treatment in pain relief (2) Severity of synovitis was not significantly related to pain relief

Flanagan/1988	Grades 1–5	NA	Positive responders: week 4: 9	Positive responders: week 8: 4; week 24: 3; week 36: 2; week 48: 1	NA	(1) Severity of OA (2) Time of the symptoms was present (3) Concentric type of arthritis	(1) The patients had good pain relief within months but not longer than 1 year (2) Patients with severer symptoms had a longer response (3) Concentric type of arthritis does not respond to IAST

Margules/2001	NA	NA	NA	Pain relief responders: week 8: severe group: 9% (21/234); moderate group: 58% (131/226); mild group: 90% (46/51)	NA	Severity of OA	(1) IAST is an efficacious and safe treatment in pain relief (2) Patients with a slighter grade severity of OA had a higher positive responder rate on pain relief

Deshmukh/2011	VAS	Positive responders: 150	Positive responders: 15–20 min: 148; week 2: 155	NA	NA	(1) Severity of OA (2) Gender and age	(1) Pain relief following IAST related to radiographic severity of OA (2) Neither gender nor age was a predictor

Robinson/2007	WOMAC pain scores	40 mg: 12 (2–20) 80 mg: 12 (2–20)	6 weeks: 40 mg: 10 (1–20) (*p* < 0.001); 80 mg: 8 (1–20) (*p* < 0.001)	12 weeks: 40 mg: 12 (1–20) (*p* > 0.05); 80 mg: 10 (1–20) (*p*=0.002)	NA	(1) Dose (2) Stiffness at baseline (3) BMI (4) Severity of OA	(1) The 80 mg dose had longer efficacy in week 12 (2) The responders had less stiffness at baseline (3) Not associated with BMI and severity of OA

Subedi/2015	OHS	NA	NA	Positive responders: 82 (all grades of osteoarthritis)	NA	Severity of OA	IAST is a highly effective therapeutic measure for hip osteoarthritis across all grades of disease severity

Walter/2019	EQ5D-VAS	0	<8 weeks: Δ in EQ5D-VAS: 1 ± 18.32 (*p*=0.915)	≥8 weeks: Δ in EQ5D-VAS: 0.25 ± 20.58 (*p*=0.455)	NA	(1) Days from injection to surgery (2) Dose, sex, short/long-term follow-up, age, and BMI	(1) No improvements in pain at short- and long-term intervals up to 6 months (2) Positive correlation with the number of days to surgery and patient-reported outcomes (3) Not related to dose, sex, age, and BMI

Young/2012	WOMAC pain scores	Low volume: 12.2 High volume: 12.3	NA	3 months' Δ in pain: low volume: 8.8 (−28%); high volume: 8.9 (−28%)	One episode of temporary hyperglycaemia in a type 1 diabetic, one facial flush, one patient reported soft-tissue swelling, and two patients reported a temporary increase in pain	Injection volume	IAST is an effective therapeutic measure across volume of 3–12 ml

NRS: numerical rating scale; NA: not available; IAST: intra-articular steroid therapy; NSAIDs: nonsteroid anti-inflammatory drugs; VAS: visual analog scale; OA: osteoarthritis; WOMAC: Western Ontario and McMaster Universities Arthritis Index; US: ultrasound; BMI: body mass index; EQ5D-VAS: EuroQol 5-domain visual analog scale; OHS: the Oxford hip score; Δ: change from baseline. ^#^The pain score data were extracted from the accompany graph in the included studies.

**Table 3:** GRADE evidence profile of pooled analysis.

Summary of findings				Quality assessment					
No. of studies	No. of patients		Effect	Design	Limitations	Inconsistency	Indirectness	Imprecision	Quality
	Steroid	Placebo							
*Hip pain*									
1-2 weeks (2)	51	55	SMD (95% CI): −1.58 [−3.42, 0.26]	RCTs	No serious limitations	No serious inconsistency^$^	No serious indirectness	Serious^*∗*^	Moderate
3–4 weeks (4)	122	116	SMD (95% CI): −1.93 [−3.34, −0.52]	RCTs	No serious limitations	No serious inconsistency^$^	No serious indirectness	No serious imprecision	High
8–12 weeks (5)	162	137	SMD (95% CI): −1.77 [−2.94, −0.61]	RCTs	Limitations^#^	No serious inconsistency^$^	No serious indirectness	No serious imprecision	Moderate

GRADE: Grading of Recommendations Assessment, Development and Evaluation; SMD: standard mean difference; RCTs: randomized controlled trials. ^#^One case-control trial was included that might raise the risk of bias. ^$^Even substantial heterogeneities were found across the included trials, and a consistent trend favoring steroid of each trial was also identified. ^*∗*^The 95% confidence interval of SMD containing the zero line might be accounted for downgrading.

## Abbreviations

IAST: Intra-articular steroid therapy
OA: Osteoarthritis
RCTs: Randomized controlled trials
THR: Total hip replacement
ACR: American College of Rheumatology
GRADE: Grading of Recommendations Assessment, Development and Evaluation
SD: Standard deviation
SMD: Standardized mean difference
ITT: Intention to treat
MP: Methylprednisolone
TAC: Triamcinolone acetonide
EQ5D-VAS: EuroQol 5-domain visual analog scale
CI: Confidence interval
VAS: Visual analog scale
WOMAC: Western Ontario and McMaster Universities Arthritis Index
OARSI: Osteoarthritis Research Society International.
